# The New Electric Tramcars

**Published:** 1889-05-18

**Authors:** 


### THE NEW ELECTRIC TRAMCARS.

There is now on view at Northfleet an electrical tramcar, which is probably quite new in this country. Everybody has felt that it would be a blessing if some substitute could be found for the jaded and overworked tramhorse, but up to the present time no suitable substitute has appeared. The steam-trams are as nightmares to other horses, and passengers are choked with sulphurous vapour and buried in smuts if they attempt a long journey on a steam-tram. It must therefore be admitted that steam is not a suitable substitute for horse-power, so far as our present knowledge goes. But there are other electrical tramcars! Certainly there are. Let us examine the different systems in use.

There are known at present three possible systems of running tramcars by electricity. The first of these is known as the accumulator system, because the cars carry charged accumulators. The second is known as the parallel system, because electricity is generated by a stationary engine and dynamo, and distributed through the cars in parallel; that is to say, the current passes through each car and back again to the generating station. The third system, and the one to which we wish to call most attention, is the "series" system, where there is only one current, which passes through the whole circuit, and is tapped, as it were, by each car.

The accumulator system can be dismissed with a very few words, as it has never met with any great success, and that for well-known reasons. The accumulators have a very considerable weight for the power that they develop, so that a car which would without the accumulator require four horse-power to propel it, will with the accumulator require six horse-power. The inefficiency is great; they give back but a very small proportion of the energy put into them, generally less than 50 per cent., and finally the depreciation on them is very great—50 per cent. per annum being the least estimate possible for this. The parallel system is undoubtedly good in many ways, and for a limited number of cars is the cheapest possible, but as the number of cars grows larger the cost of working them is enormously increased.

The series system avoids the disadvantage of the parallel, as the more cars there are on the line the less is the loss per cent. We will briefly examine the reason of this, and then pass on to explain the working of the series system. We have said before that in the parallel system the current passes out from the dynamo along one conductor, through the car, and back again to the power by the other conductor. For each car put on the road, therefore, an additional current must be sent along the conductor. Two disadvantages result from this—namely, great loss of power owing to the amount of electricity in the conductors, and great increase of work. It is known that the work required is directly proportional to the square of the current quantity, hence the work required to send two currents is four times as great as to send one, nine times as great for three, sixteen times as great for four, and so on, and it now becomes quite obvious to every-one why the cost increases so enormously as the number of cars, in the parallel system, increases. Consequently the parallel system could never be adopted to a large extent, either the cost of running would be too great, owing to this loss of power in conductors, or else a very large sum would have to be expended in the conductors to keep the loss down to a reasonable figure. In the series system the current on the line is a constant quantity. It does not increase as the number of cars increases, and the electromotive force varies directly with the number of cars on the line. On the side of expense, then, the new system is superior to the old ones.

The whole principle of the system is that the same current is made to pass through every car on the circuit. This is attained by means of two contrivances called the spring-jack and the current collector. The conductor is cut into sections of 21ft. in length, each section having a little bridging spring connecting it with the bridging spring of the next section, and so on round the circuit. The bridging springs are the spring-jacks, and present much the appearance of two half-closed hands placed back to back. This conductor is placed almost immediately under one rail of the tramway, and the rail, instead of being solid throughout as an ordinary rail, has a slot cut in the very centre. The current collector is a long rod of insulating material with copper or brass strips fastened to its sides. It is below the roadway, and is supported from the car by four iron arms which are bent so as to bring the current collector exactly to the level of the spring-jacks. Now, suppose the car to be starting; the current collector is twenty-six feet long, so that it is always in connection, and keeping at least one spring-jack open. The current cannot pass through the insulating material of the collector, and is bound, therefore, to follow the connections of the strip of metal along its side, and be led through the motor of the car. The car at once begins to move, dragging its arrow with it beneath the roadway. As the collector is twenty-six feet long, before the first set of spring-jacks are passed, and while the current is still passing by their means through the car, and thence along the line, the front part of the collector is pushing open another set of spring-jacks, and the current is in turn led by these through the motor which is carried by the car. The electric force consequently is always acting and the car moving, unless the driver breaks the connection, in which case the car will stop when its energy is lost. More than this, there is an apparatus attached by which the driver can at once reverse the gearing and put on a powerful break and stop in half the length of the car, a thing that can be done in no other locomotive engine in the world. It is scarcely necessary to go into the question of the road bed as the only apparent difference is the slot in the one line, and the hollow place beneath it in which are the spring-jacks and collectors. There is also an arrangement by which some of the current is diverted to lighting the cars with electric light. Such, then is a brief account of the working and system of the series trams. It appears logical and reasonable on the face of it, and it is our firm belief that the Series Electrical Tramway system has a large future before it.

